# Modeling the effect of prolonged ethanol exposure on global gene expression and chromatin accessibility in normal 3D colon organoids

**DOI:** 10.1371/journal.pone.0227116

**Published:** 2020-01-17

**Authors:** Matthew Devall, Lucas T. Jennelle, Jennifer Bryant, Stephanie Bien, Ulrike Peters, Steven Powell, Graham Casey

**Affiliations:** 1 Center for Public Health Genomics, Department of Public Health Sciences, University of Virginia, Charlottesville, Virginia, United States of America; 2 Division of Public Health Sciences, Fred Hutchinson Cancer Research Center, Seattle, Washington, United States of America; 3 Digestive Health Center, Gastroenterology and Heaptology, University of Virginia, Charlottesville, Virginia, United States of America; Singapore General Hospital, SINGAPORE

## Abstract

In this study we aimed to explore the potential biological effect of ethanol exposure on healthy colon epithelial cells using normal human colon 3D organoid “mini-gut” cultures. In numerous published studies ethanol use has been shown to be an environmental risk factor for colorectal cancer (CRC) development; however, the influence of ethanol exposure on normal colon epithelial cell biology remains poorly understood. We investigated the potential molecular effects of ethanol exposure in normal colon 3D organoids in a small pilot study (n = 3) using RNA-seq and ATAC-seq. We identify 1965 differentially expressed genes and 2217 differentially accessible regions of chromatin in response to ethanol treatment. Further, by cross-referencing our results with previously published analysis in colorectal cancer cell lines, we have not only validated a number of reported differentially expressed genes, but also identified several novel candidates for future investigation. In summary, our data highlights the potential importance for the use of normal colon 3D organoid models as a novel tool for the investigation of the relationship between the effects of environmental risk factors associated with colorectal cancer and the molecular mechanisms through which they confer this risk.

## Introduction

Colorectal cancer (CRC) was attributed to over 50,000 deaths in 2018 and ranks as the third most common cancer among both men and women across the United States [1]. While genome-wide association studies (GWAS) continue to identify a growing number of genetic variants associated with CRC risk [2], multiple studies have also implicated a role for environmental exposures [3]. For example, a comprehensive evaluation of the literature conducted by the World Cancer Research Fund found evidence of a strong association between the consumption of two or more drinks of alcohol per day and an increase in CRC risk [4]. Despite this, understanding of the effect of alcohol on normal colon epithelial biology and how it may lead to CRC remains elusive.

Here, we conducted a pilot study to interrogate the effect of prolonged alcohol exposure using a 3D normal colon organoid model system and next generation sequencing approaches at the transcriptomic (RNA-seq) and chromatin (ATAC-seq) levels. Normal epithelial colon organoids derived from healthy patients [5, 6] provides a novel model to the study of both environmental and (epi)genetic factors affecting the normal colon and colon cancer development. The 3D structure of the organoid resembles that of the colon, encompassing much of the cellular complexity of the epithelia of the colonic crypt from which it was derived and as such, has been coined the “mini gut” [7]. Indeed, a number of studies have supported the utility of colon organoids as a model system by determining that epigenetic modifications present within organoids are representative of those within the colon epithelium [8, 9] and providing valuable insight into crypt biology. Further, the utility of this model in the assessment of an environmental risk factor of CRC (i.e. high fat diet) revealed that treatment of 3D organoids with components of a high fat diet led to similar functional and morphological changes as those identified in the intestines of mice fed on a high fat diet, highlighting the potential of this model in environmental exposure studies [10].

RNA-seq has been employed extensively to correlate changes in gene expression with specific perturbations [11, 12]. ATAC-seq [13] also has the potential to reveal changes in the regulatory landscape of the genome, and has been found to be influenced by factors such as disease state [14]. Further, changes to enhancer profiles have been shown to be highly correlated with changes in the expression of relevant genes [15]. By using a multi-omics approach we sought to gain a greater understanding of the transcriptomic and epigenetic basis of ethanol exposure in order to identify novel molecular pathways affected by ethanol exposure that may lead to a dysregulation in normal colon epithelial biology. Using this multi-omics approach, we identified a number of genes associated with ethanol exposure, including ones previously reported, and highlight novel mechanisms that may underly the role of ethanol in CRC risk and development.

## Materials and methods

### Patient population and sample demographics

Subjects undergoing routine colonoscopy through the period of July–October 2017 were enrolled were enrolled under an approved Institutional Research Board for Health Sciences Research of the University of Virginia after providing informed consent. For the purpose of this pilot, only Caucasian males were enrolled into this study between 34–59 years of age (34, 53, 59 years of age). Colon biopsies were taken from the right side, just proximal to the hepatic flexure. Samples were collected from patients during a routine surveillance colonoscopy at the Digestive Health Centre of the University of Virginia and only patients presenting with an absence of personal and family history of CRC as well as no colon related pathology were selected. Within reason, given the small sample size, these samples could be considered as representative of a wider population of healthy inidividuals undergoing routine colonoscopy. All methods were performed in accordance with the relevant guidelines and regulations and were consistent with those required by both the NIH and the University of Virginia. This study was approved by the Institutional Review Board of the University of Virginia.

### Colon epithelial 3D organoid cultivation and ethanol exposure

Colon biopsies were obtained after routine colonoscopy and established as viable, normal colon 3D organoids using a modification of the method described by Sato et al [16]. After isolation, colon crypts they were embedded in Matrigel and grown in growth media that included: advanced DMEM/F-12, 100U/ml penicillin, 100μg/ml streptomycin, 10mM Hepes, 1x N2, 1x B27, 2mM GlutaMAX, 1mM N-acetyl-cysteine, 10mM gastrin, 50% L-WRN conditioned media, 500nM A83-01, 10uM SB202190, 10mM nicotinamide, 50ng/ml EGF, 10μM Y27632. After colon 3D organoid establishment, lines were fed and passaged as required. For this experiment, cells were treated with fresh growth media plus ethanol (2 μl (Sigma Aldrich; Cat No: E7023) per 1ml of growth media, 2μl:1000 μl or 0.2% vol:vol [43mM]), or fresh growth media plus 2 μl distilled water as a control. Media (with or without ethanol) was replaced every 24-hours for 3 days in order to replace evaporating ethanol and ensure consistent levels of exposure throughout the experiment. This method has been chosen to better reflect blood alcohol levels of regular drinkers. In our protocol a single well of a 48-well plate of organoids typically contained ~0.25–0.3x10^6 cells after 72 hours. Thus, each well corresponded to ~0.25–0.3 x10^6 organoids exposed to 1uL Ethanol in a Total of 500uL (2uL/mL, 0.2%) Complex Media. To obtain >1ug RNA ~3 wells were used equating to approximately 0.75-1x10^6 cells total. The ethanol dose was chosen after a thorough literature search identified similar ethanol treatment protocols in numerous cell lines including relevant human colon-derived cell lines [17, 18].

### 3D organoid imaging

At the time of the study all organoid lines were between passage 7 and 15, and images were taken throughout the exposure to determine health, morphology and viability remained consistent throughout. Images of the three [3] independent 3D colon organoid lines used in this study captured during routine culture (**S1 Fig**). Images were captured from a CKX53 Cell Culture Microscope (Olympus) using an Infinity2 USB Microscope Camera and processed with Infinity Analyze and Capture software (Lumenera).

### RNA-sequencing and data processing

For RNA isolation, media was removed gently insuring that the Matrigel containing the organoids was not disturbed and 200 μl RNA lysis Solution RA1, without TCEP (Machery-Nagel; Cat No: 740902.50) was applied to the entire Matrigel dome lysate, which was collected in a 1.5ml Eppendorf tube. Each tube was vortexed briefly and placed on ice. Further steps were carried out according to manufacturer’s protocol of the Machel-Nagel RNA XS kit. RNA was quality checked using the Agilent 4200 Tapestation and only samples with a RIN score greater than 9.8 were for library preparation and sequencing using the Illumina HiSeq 4000 (Northwest Genomics Center at the University of Washington, Seattle). Briefly, 100bp paired-end reads were quality checked; low quality reads and adaptors were then trimmed and the resulting sequences were aligned to the latest reference transcriptome (GRCH38) using STAR (v.2.5.3a) [19]. An average of ~85 million reads mapped per sample, representing a mean per base coverage of 44.62 million reads and an average mapping efficiency of 85.52%. Resulting BAM files were then indexed and marked for duplicates using the Picard MarkDuplicates tool. Gene-level counts were then determined using the featureCounts tool in the RSubread package [20] and differential expression analysis was carried out using DESeq2 [21].

### ATAC-seq library preparation and analysis

ATAC-seq was performed using a modified version of Omni-ATAC [22]. To break down the Matrigel, organoids were subjected to treatment with Accutase (Sigma-Aldrich; Cat No: A6964-100ML). Post treatment, samples were centrifuged at 300 x g for 5 minutes, cells were counted and processed using the recommended protocol for cell culture [22] with the following exceptions: to account for the small size of the cells, all subsequent centrifugation steps were performed at 1000 x g for 10 minutes at 4°C; following the removal of the last 100 μl of ATAC-seq resuspension buffer, a second cetrifugation and supernatant removal was performed, which was found to improve Tn5 efficiency. Following sample preparation, samples were sent for sequencing on the Illumina HiSeq 4000 (Northwest Genomics Center at the University of Washington, Seattle). Each sample generated approximately 50 million, 75bp paired end reads. Samples were quality checked and trimmed for reads of low sequence quality and adapters. Samples were then aligned to the Gencode reference Genome GRCH38.p13 using bowtie2 [23]. An average mapping efficiency of 45.83% reads mapped to the human reference genome; after removal of duplicates and mitochondrial reads, an average of ~23 million ATAC-seq reads were analyzed. BAM files were indexed and marked for duplicates using the Picard MarkDuplicates tool. BAM files were further analyzed according to ENCODE specifications. Peaks were then called using MACS2 PeakCaller [24] with the following parameters: ––nomodel ––shift 37 ––extsize 73 ––broad –keep-dup all –B. A consensus peak set of 70,824 peaks was then generated using DiffBind (v2.8.0) [25] and nominally significant peaks (p = 0.05) were generated using DESeq2 library normalization procedures.

### Quantitative PCR design and analysis

Gene expression validation experiments were performed on RNA isolated from an independent set of the same 3D organoid lines exposed/unexposed to ethanol. RNA for quantative PCR (qPCR) was isolated essentially as described in RNA-Sequencing and Data Processing. RNA concentration was determined on a Qubit fluorometer (Thermo-Fisher). One microgram of Total RNA was Reverse Transcribed to first-strand cDNA using the High-Capacity cDNA Reverse Transcription Kit (Thermo-Fisher). First-Strand cDNA was used for Taq-Man qPCR monitored on a QuantStudio Real-Time PCR analyzer (Thermo Fisher). Pre-Designed TaqMan Gene Expression Assays (Thermo Fisher) were used for quantification of *ANPEP*, *ARHGAP32*, *KIF16B*, *GNL3*, *ECE1* and the control gene *HPRT1*: Hs00174265_m1, Hs00206951_m1, Hs00402541_m1, Hs00205071_m1, Hs01043735_m1 and Hs02800695_m1 respectively.

## Results

### First transcriptome-wide analysis of ethanol exposure in normal colon 3D organoids identifies enrichment for CRC-related genes

**Fig 1:**
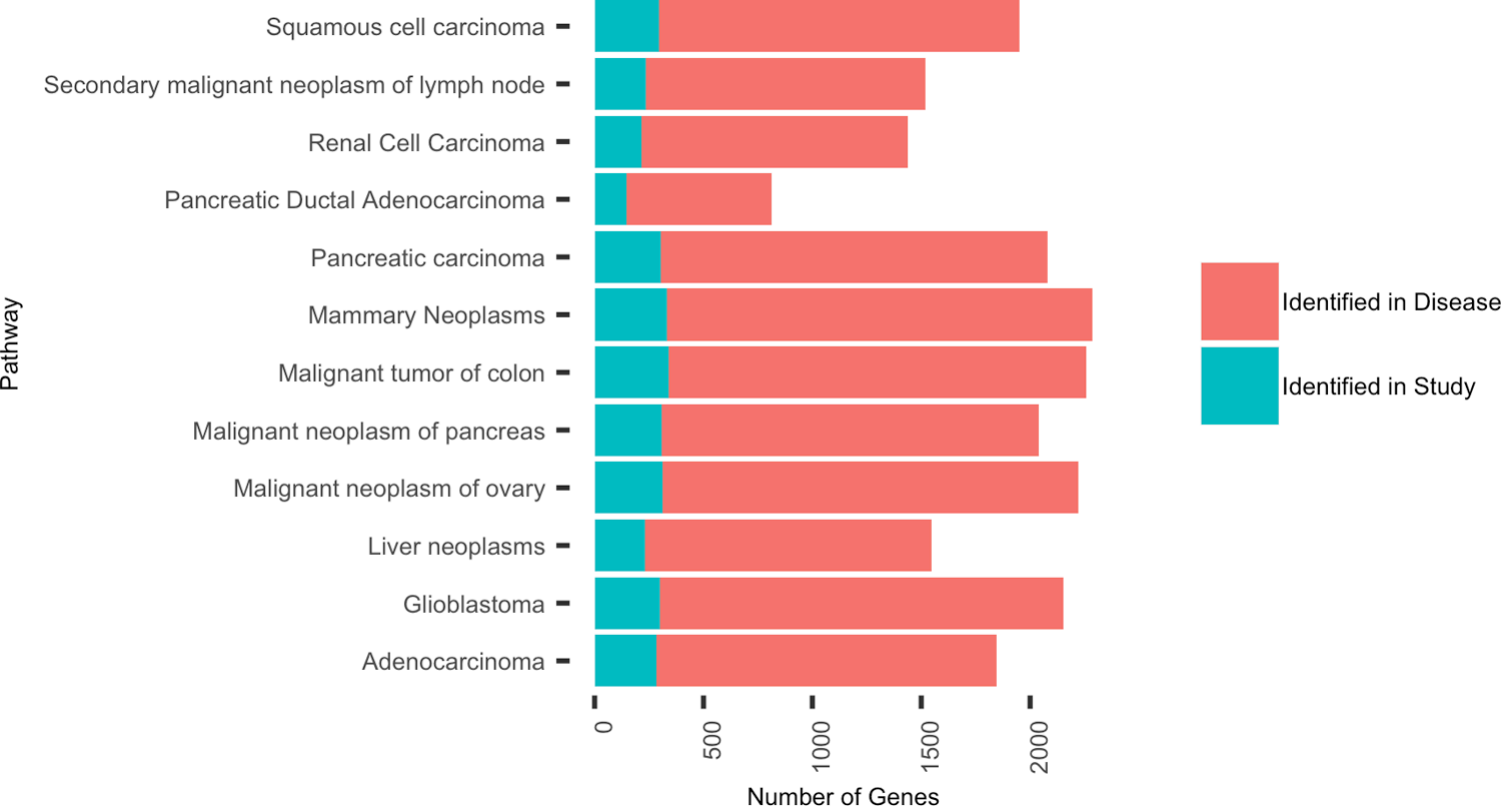
Differential expression analysis shows enrichment for multiple relevant pathways. For all fdr corrected differentially expressed genes, an enrichment analysis was performed using ToppFunn, and relevant pathways were plotted.

Following alignment to the human reference genome, gene count summation and removing genes of low count (**see** Methods) 14,762 genes remained for differential expression analysis in DESeq2 [21], of which 1,965 were found to be significantly differentially expressed in colon organoids (q = 0.05) in response to ethanol treatment. For example, a number of the DEGs identified here are known to be involved in the metabolism of alcohol and its byproducts: such as *Aldehyde Dehydrogenase 3 Family Member B1* (*ALDH3B1*; q = 1.30E^-11^), *ALDH2* (q = 1.12E^-05^) and *ALDH1L1* (q = 2.62E^-05^). Of these 1,965, 1,360 genes were found to be downregulated in ethanol treated organoids (**Table A in S1 File**). A Fisher’s exact test was performed, which showed that this pattern of fold change in our significant differentially expressed genes (DEGs) represented a highly significant enrichment (p < 2^E-16^, odds ratio = 2.566) for downregulation of gene expression with respect to the overall distribution of expression between the two conditions, suggesting that these genes may be involved in similar pathways. To determine the biological relevance of these genes, we tested whether they were enriched in genes associated with drugs and disease using the ToppFun function of the ToppGene suite [26]. Importantly, this analysis determined that DEGs in our study were associated with ethanol (q = 2^E-28^) and also with multiple colon related pathways such as malignant tumor of colon (q = 1^E-23^) and adenocarcinoma (q = 2^E-19^). Indeed, of the 1,965 genes used for this annotation, 342 were found to be associated with malignant tumor of colon (**Fig 1 and Table B in S1 File**). Further, we performed qPCR validation analysis and found that of the five genes tested: *ANPEP*, *ARHGAP32*, *KIF16B*, *GNL3* and *ECE1*, all but *GNL3* were differentially expressed in a direction consistent with RNA-Sequencing (**S1 Fig**).

### Ethanol exposure of colon 3D organoids lead to widespread changes in chromatin accessibility identified using ATAC-seq

**Fig 2:**
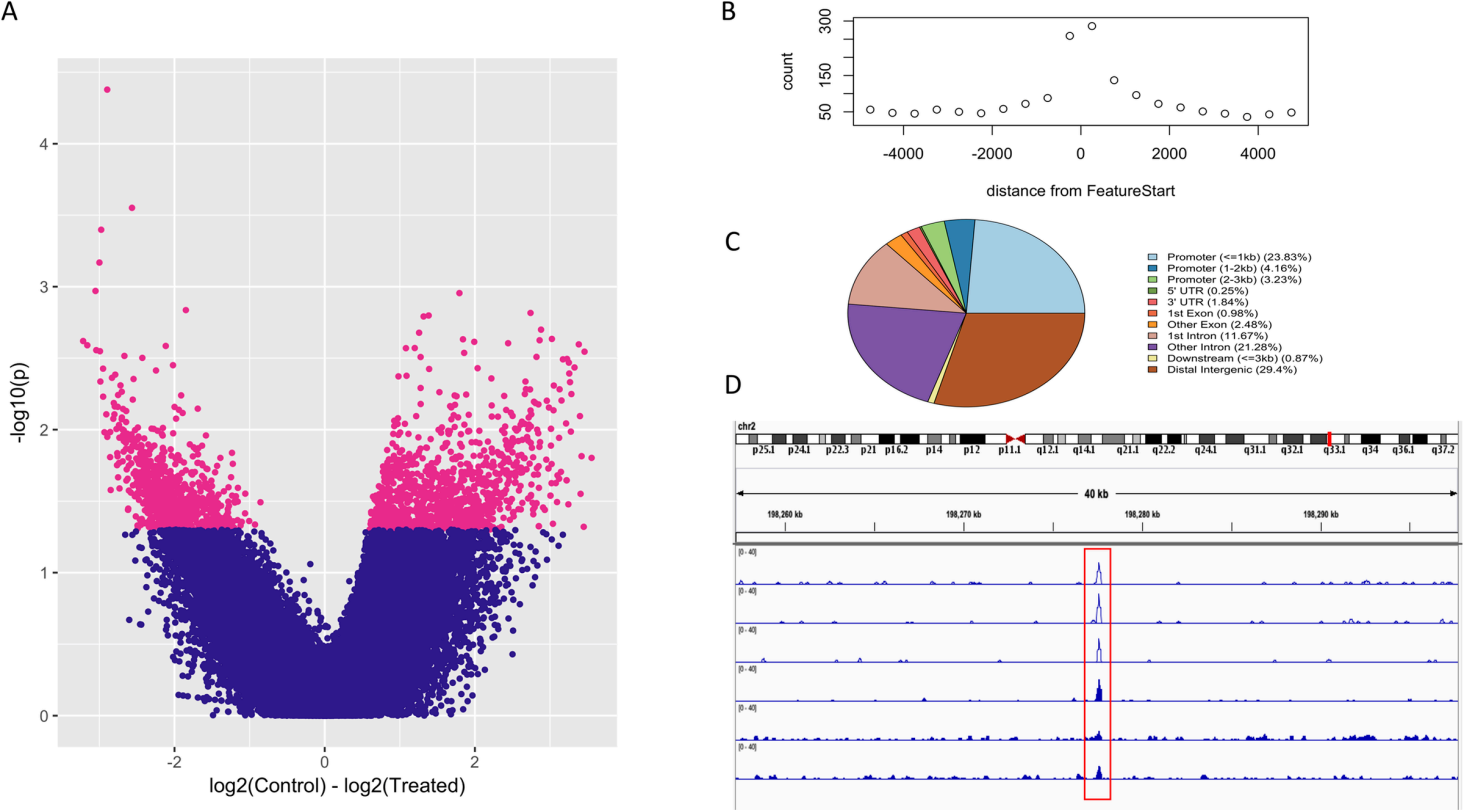
ATAC-seq analysis. **(A)** Volcano plot to show differentially accessible peaks, nominally significant peaks (p = 0.05) and shown in pink. **(B)** Annotation of an example peaks shows increased counts at transcription start sites. **(C)** Distribution of all peaks in the consensus peak set across genomic features. **(D)** Example track of ATAC-seq peaks, ethanol treated samples are shaded in blue.

To determine whether gene expression changes were likely due to variation in chromatin accessibility brought about by ethanol exposure, we adapted Omni-ATAC [22] for use on matched colon organoid samples previously examined using RNA-Seq (**see** Methods). Of the 70,824 consensus peaks, 2,216 were found to be differentially accessible (p = 0.05) in association with ethanol exposure (**Fig 2A–2D and Table C in S1 File**), 1,498 of which were annotated to a total of 1431 nearby genes. To gain a further understanding of the relationship between these differential peaks, an enrichment analysis was again performed using the ToppFun function of ToppGene; this identified a nominally significant enrichment of differential peaks associated with malignant tumor of colon (q = 8^E-02^).

### Investigating the relationship between the two omic layers

**Fig 3:**
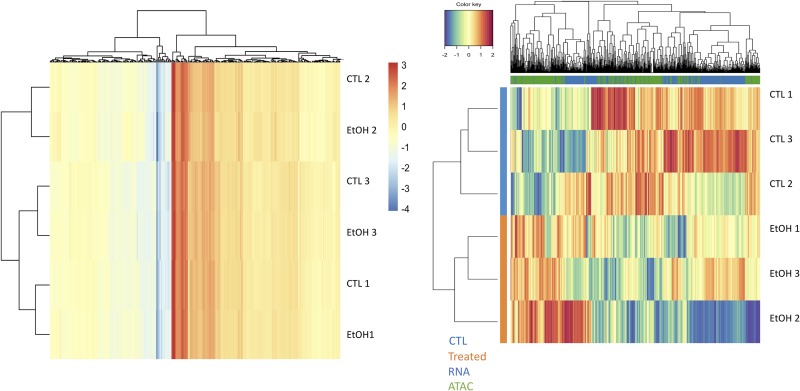
Clustering analysis of differentially expressed genes and differentially accessible regions. **(A)** Hierarchical clustering of normalized counts of differentially expressed genes led to partial clustering of treatment response. **(B)** Hierarchical clustering of 114 normalized gene counts and differentially accessible peaks for genes found to be significant across both omic layers led to complete separation of samples based upon treatment response. Increasing values indicated increased gene expression or chromatin accessibility in controls relative to ethanol treated samples.

Of the 1,965 significant DEGs, 152 were mapped near to 158 differential peaks identified by ATAC-seq (**Table D in S1 File**). There was a significant enrichment for Malignant Tumor of Colon (q = 1.47^E-4^) in this small subset of genes using ToppFunn (**Table E in S1 File**). This suggests that, by using multiple omics approaches to fine-tune data analysis, one can infer potentially relevant subsets of data for further analysis. Further, using the R package mixOmics [27] we determined that differential levels of expression and chromatin accessibility at these regions were strong enough to cluster samples according to ethanol treatment, while this was not possible using gene expression data alone (**Fig 3A and 3B**).

## Discussion

To our knowledge, this pilot study represents the first attempt to elucidate potential molecular mechanisms underlying exposure to ethanol, a consistent risk factor of CRC, using human colon epithelial 3D organoids derived from healthy individuals. We highlight several DEGs and changes in chromatin accessibility that occur in response to ethanol treatment.

We identified 1,965 differentially expressed genes that passed Benjamani-Hochberg correction for multiple testing (q = 0.05) in association with alcohol exposure in colon organoids. Several of these genes had previously been identified in alcohol exposure-related studies. For example, we identified a significant downregulation of *CDH1* (q = 2.098^E-10^), a gene also identified in a previous study of ethanol exposure in CRC cell lines [28]. Further, our study also identified a subset of genes reported as being differentially expressed in a recent study in intestinal organoids of mice exposed to ethanol treatment [29] with the same direction of effect (q = 0.1). Of the reported genes, five genes, (*UVSSA*, *MICAL2*, *ZBED6*, *MACF1* and *MLX*), were downregulated, and six genes (*HSPA14*, *SMARCAD1*, *FARSA*, *UBE3C*, *PSMD6* and *RPL7*), were found to be upregulated upon ethanol treatment in our study. Finally, pathway analysis of the significant DEGs identified in our study showed an enrichment for genes involved in both ethanol exposure and colon malignancies, providing further evidence that our pilot study was able to lead to the identification of exposure- and disease-relevant genes. For example, a number of the DEGs identified here are known to be involved in the metabolism of alcohol and its byproducts: such as *ALDH3B1*, *ALDH2* and *ALDH1L1*.

To determine how ethanol exposure may drive changes in chromatin accessibility, and thus drive differential gene expression, we performed ATAC-seq on the same normal colon 3D organoid lines. We identified 2,216 nominally significant, differentially accessible chromatin changes associated with ethanol treatment, of which 1,498 could be annotated to nearby genes. A subset of these chromatin changes mapped adjacent to DEGs, suggesting that these changes in chromatin accessibility were partially responsible for the observed change in differential expression. It is important to note that while using nearby gene annotations for enhancers and promoters, it is possible that regions of open chromatin may be acting in trans and as such, could be underrepresented in our dataset; however, without the use of chromosome conformation capture techniques such as Capture-Hi-C, the relationship between these regions and their interactions with nearby genes can only be inferred. Further, whilst one may expect a stronger overlap between differentially expressed genes and differential chromatin accessibility, low levels of overlap between chromatin architecture and gene expression have also been reported in other studies [30]. Importantly, we performed a pathway analysis of significant open chromatin peaks and identified an enrichment in genes associated with malignancy of colon, suggesting that ethanol exposure of organoids does indeed drive a differential regulation associated with colon cancers.

A strength of combinatorial multi-omics analysis is the ability to determine consistent changes across different omic layers, as seen by our pathway analysis. Here, despite the small subset of genes and overlapping chromatin regions being identified, this subset was strongly associated with malignant tumor of colon. Importantly, within this subset we identified a number of genes not previously associated with either ethanol exposure or CRC, including *KIF16B*, *ARHGAP32* and *SUB1*. *ARHGAP32* in particular is an interesting finding, given that it is a gene hypothesized to play a role in regulating both RAS- and Rho- signaling pathways [31], which have often been found to be perturbed in cancers, including CRC, as reviewed in Saeed et al., 2019 [32]. Importantly, we were also able to cluster samples according to ethanol response when a combined analysis was performed of DEGs and differential chromatin accessibility sites that overlapped these genes, while this was not possible using gene expression data alone. Finally, this data suggests that ethanol exposure could, in part, be driving gene expression changes by altering chromatin states.

In an attempt to validate our analysis we performed quantitative PCR (qPCR) assays for five genes. *ANPEP* was chosen as the most significant DEG in our dataset, whilst *KIF16B*, *ARGHAP32*, *ECE1* and *GNL3* were highly significant DEGs and were also genes associated with nearby differential accessibility. Of these five genes, we observed a downregulation of *ANPEP*, *ARHGAP32*, *ECE1*, *and KIF16B* in response to ethanol treatment in our qPCR analyses, consistent with RNA-Seq. We were unable to validate changes in *GNL3*, and this could be due to a number of technical reasons such as varying ability to capture transcript abundance across the methods.

There are four major limitations to our study. First, a single ethanol dose and a single 72-hour time period was used in this experiment. Whilst a number of studies have been published using a similar ethanol dose in CRC cell lines [28, 33] a time course experiment may reveal more nuanced gene expression and epigenetic changes associated with ethanol exposure. Further, while epigenetic modifications may occur shortly after exposure, here, we aimed to investigate the longer term effects of ethanol exposure on colon organoids in an attempt to mimic those of more prolonged alcohol use in the patient population. Second, our sample size is small. Despite this, we identified several DEGs that had previously been implicated in cell line, population-based or animal studies, supporting the veracity of our analysis. Third, we limited our study to males only, due to the pilot nature of the study, and caution should be taken when inferring relevance of these findings to females and indeed, to non-Caucasian males. Expanding our study to a larger, more diverse sample population has the potential to identify additional novel DEGs related to ethanol exposure. Fourth, while RNA-seq is the current gold-standard for transcriptome-wide analysis, averaging of signal across a heterogenous cell population can lead to difficulties with interpretation of results. The use of specialized complex media aims to minimize this effect by maintaining the majority of cells in an undifferentiated state. However, in the future, exposure studies across isolated cell populations or indeed, those using single-cell RNA-seq technologies may serve to improve the resolution of the changes observed in this analysis.

In summary, despite the limited sample size of our study, we were able to validate a number of genes previously identified with ethanol exposure and/or CRC as well as identify a number of novel candidates for future investigation. We also show an enrichment of pathways related to alcohol exposure and colon cancer in both omic analyses, suggestive of biological relevance. These results suggest that the normal colon 3D organoid model may represent a powerful tool for the evaluation of epidemiological risk factors associated with CRC.

## Supporting information

S1 Fig 3D organoid image at 72 hour post exposure. For each individual, images were collected at the end of the exposure to assess organoid morphology.
(TIF)

S2 Fig QPCR validation of differentially expressed genes. Of the five genes selected, all but GNL3 were found to follow the same direction of effect as in the RNA-Seq analysis. Relative gene expression for each gene was calculated against the control gene HPRT1. Increase expression in ethanol treated samples can be seen as a value greater than 1.
(TIF)

S1 File Summary tables of differential expression and differential chromatin accessibility analysis. All pathway analysis was performed using Toppfun. Negative fold changes are associated with increased accessibility or expression in ethanol treated samples.
(ZIP)
