# Supplementary figures and images for: Modeling the effect of prolonged ethanol exposure on global gene expression and chromatin accessibility in normal 3D colon organoids
Source: PLoS One. 2020 Jan 17;15(1):e0227116. doi: 10.1371/journal.pone.0227116 (PMC6968849; doi:10.1371/journal.pone.0227116)

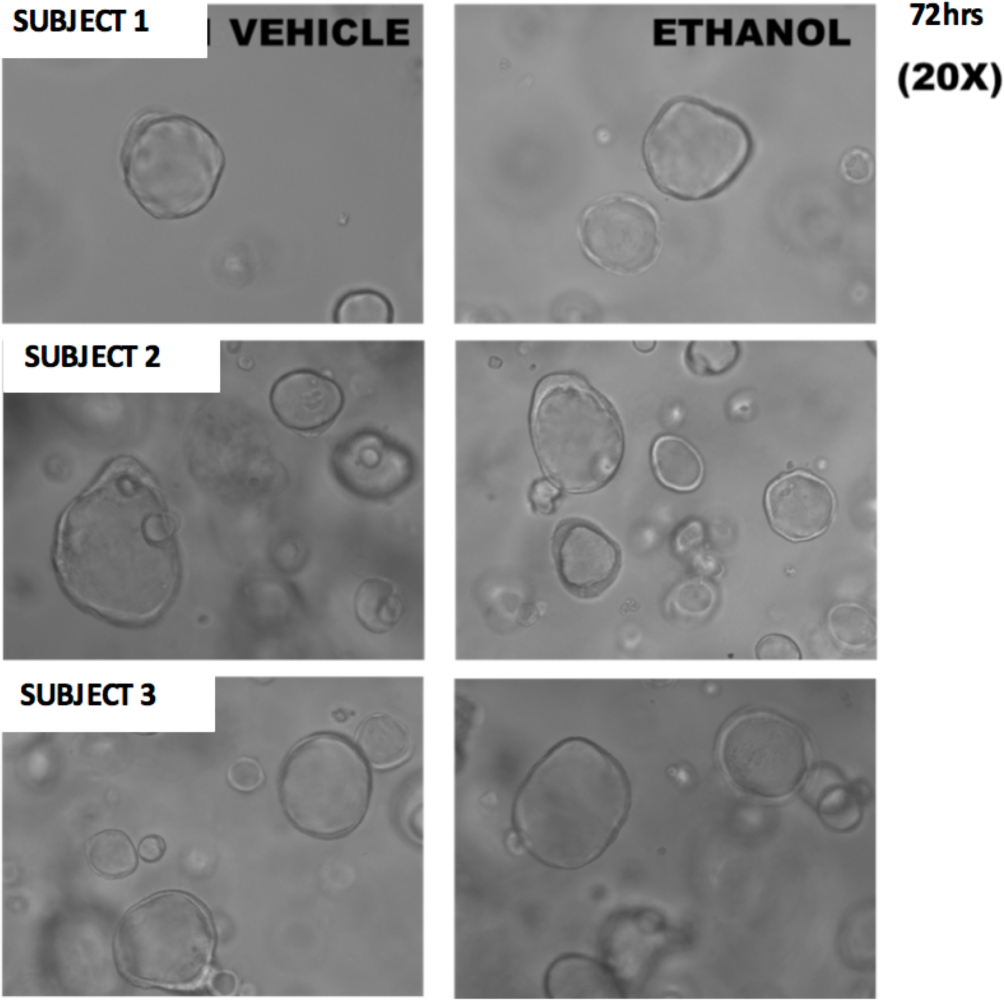

Supplement: S1 Fig — For each individual, images were collected at the end of the exposure to assess organoid morphology. (TIF) [file pone.0227116.s001.tif]

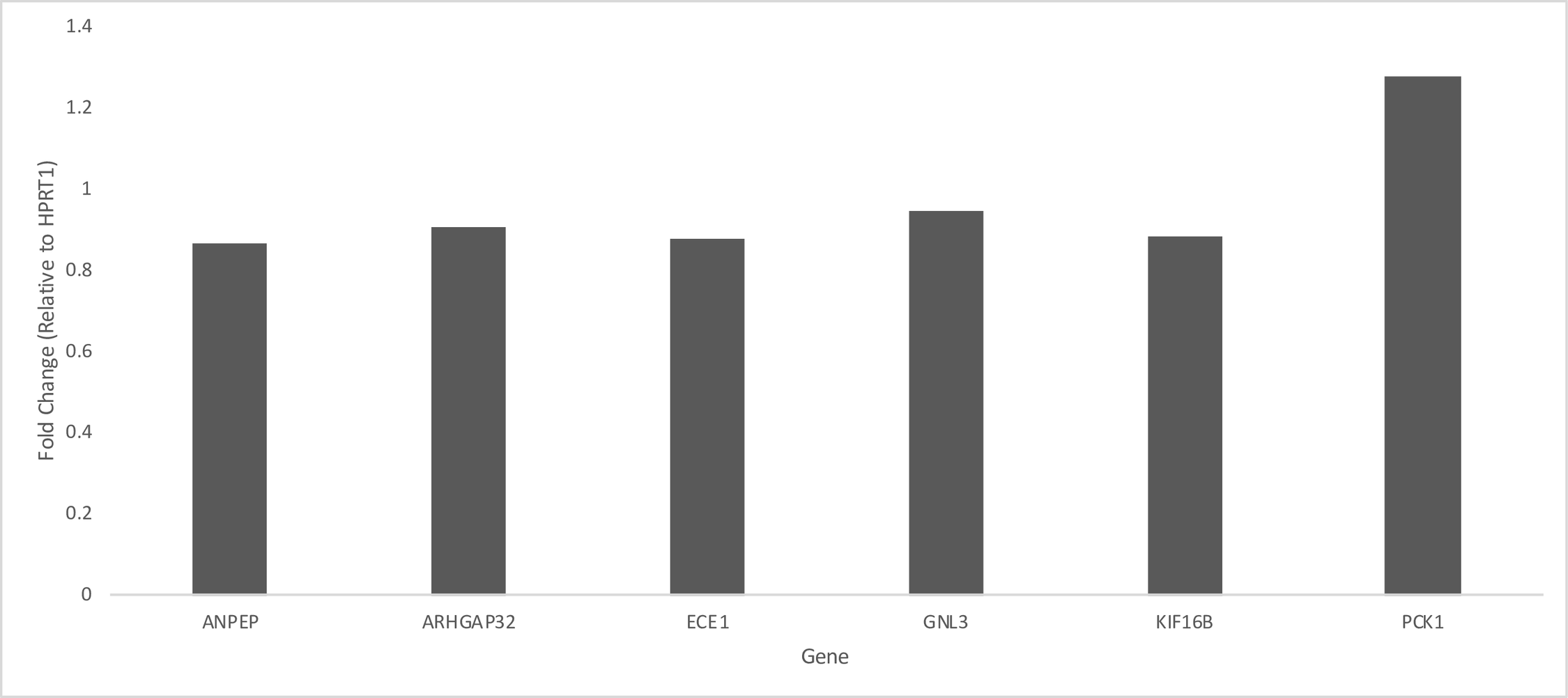

Supplement: S2 Fig — Of the five genes selected, all but GNL3 were found to follow the same direction of effect as in the RNA-Seq analysis. Relative gene expression for each gene was calculated against the control gene HPRT1. Increase expression in ethanol treated samples can be seen as a value greater than 1. (TIF) [file pone.0227116.s002.tif]
